# Therapeutic exercise versus other modalities for prevention and treatment of low back, pelvic girdle, and lumbopelvic pain during pregnancy: A review protocol

**DOI:** 10.1371/journal.pone.0274471

**Published:** 2022-09-22

**Authors:** Silvia Oliveira Ribeiro Lira, Vanessa Patrícia Soares de Sousa, Elizabel de Souza Ramalho Viana

**Affiliations:** 1 Department of Physical Therapy, Universidade Federal do Rio Grande do Norte, Natal, Rio Grande do Norte, Brazil; 2 Universidade Federal do Rio Grande do Norte, Faculdade de Ciências da Saúde do Trairi (FACISA/UFRN), Santa Cruz, Rio Grande do Norte, Brazil; Flinders University, AUSTRALIA

## Abstract

**Background:**

The female body changes during pregnancy to create a favorable environment for fetal development which may result in musculoskeletal disorder and painful symptoms in the lumbopelvic region.

**Objective:**

To analyze the evidence of therapeutic exercise *versus* other modalities to prevent and treat LBP, LGP, and LPP during pregnancy.

**Methods:**

Full text randomized controlled trials (RCT) evaluating interventions to prevent or treat LBP, PGP, and LPP during pregnancy (any gestational age) that comparing therapeutic exercises with usual care or other modalities to reduce the incidence or severity of LBP or PGP or both during pregnancy will be included. 5 electronic databases will be searched to identify studies. Assess risk of bias in each study using the Cochrane Handbook for Systematic Reviews of Interventions and quality of overall body of evidence for all primary outcomes will be assessed for all comparisons using the approach outlined in GRADE Handbook.

## Background

### Description of the condition

The female body changes during pregnancy to create a favorable environment for fetal development which may result in musculoskeletal disorders [1, 2] and painful symptoms [3], especially in the lumbopelvic region [4].

Mechanical and hormonal factors may cause morphophysiological changes during pregnancy and induce dysfunctions in the lumbopelvic region [5]. The lumbopelvic pain (LPP) includes low back (LBP) or pelvic girdle pain (PGP) or both and is the most common [1], severe, and incapacitating complaint [6] during pregnancy.

Approximately 10% to 21% of pregnant women report severe and incapacitating LPP. Moreover, 50% to 70% of pregnant women present LBP and 10% to 65% have PGP [7]. LPP impacts quality of life [8], causing functional disability [9] and affecting activities of daily living [1]. Also, some pregnant women present pain reduction after childbirth, while 5% to 8.5% report pain up to two years after childbirth [10].

### Description of the intervention

Non-pharmacological treatment for LBP, PGP, and LPG during pregnancy consists of ergonomic modifications, resting periods, hot and cold compresses, support belts, massage, acupuncture, yoga, manipulative practices, and pregnancy-specific exercises [11]. Regular physical activity of moderate-intensity for at least 20 to 30 min per day on almost all days of the week may also protect against the development of LPP during pregnancy [12]. Therefore pregnant women without medical or obstetric complications should be encouraged to perform exercises [13, 14] since low levels of physical activity may affect muscle function and the development of lumbopelvic pain in this population [8, 15].

Therefore, this review protocol will consider studies analyzing therapeutic exercises to prevent and treat LBP, PGP, and LPP during pregnancy.

### How the intervention will work

Exercise is a planned, structured, and repetitive physical activity for body conditioning [8] that has greater positive effect on severity of LBP than usual care [16]. Exercises for pregnancy-related LBP are similar to those for non-specific LBP [17]. For example, walking, low-impact aerobic exercise, and adapted yoga and pilates are safe physical activities for pregnancy [13].

Regular physical activity during pregnancy promotes health benefits, such as decreased frequency of gestational diabetes mellitus, prevention of preeclampsia, and improved recovery time during postpartum [13]. Exercise may also reduce pain intensity and disability and improve global functioning [14, 18].

### Why is this review important?

Pregnancy-related LPP impacts daily functioning and well-being and is treated by physiotherapists using passive and active treatments (e.g., mobilization and exercise, respectively). However, consensus or guidelines about the best modality or type, duration, and frequency of exercise for LPP during pregnancy are not yet available. Furthermore, previous reviews did not analyze the impact of these modalities considering functioning of pregnant women.

### Objective

This study aims to analyze the evidence of therapeutic exercise *versus* other modalities to prevent and treat LBP, LGP, and LPP during pregnancy in pain intensity (pain level), Low back or pelvic-related functional disability, functioning and quality of life.

## Methods

### Protocol and guidelines

The search strategy and reporting of this systematic review will be adhered to the PRISMA guidelines and will be followed the Cochrane group’s recommendations. The protocol will be registered in PROSPERO and we will intended search in July 2022 a may 2023.

### Type of studies

Full text randomized controlled trials (RCT) evaluating interventions to prevent or treat LBP, PGP, and LPP during pregnancy, that analyze the impact on pain intensity, functioning and quality of life.

### Type of participants

Studies including pregnant women (any gestational age) who reported, were clinically diagnosed using specific tests, or were at risk of developing LBP or LGP or both.

### Type of interventions

Studies comparing therapeutic exercises with usual care or other modalities to reduce the incidence or severity of LBP or PGP or both during pregnancy.

### Type of outcome measures

#### Primary outcomes

The following outcomes (measured using validated tools) will be included:

1. Pain intensity (pain level);
2. Low back or pelvic-related functional disability (mensured by the evaluation of validated questionnaires);
3. Functioning (mensured by the evaluation of validated questionnaires);
4. Quality of life (mensured by the evaluation of validated questionnaires);
5. Adverse events.

#### Electronic searches

Studies from the following databases and trial registries will be identified:

1. Cochrane Central Register of Controlled Trials (CENTRAL), via Cochrane Register of Studies and with no restriction regarding year;
2. MEDLINE;
3. Embase;
4. US National Institutes of Health, Ongoing Trials Register, ClinicalTrials.gov (www.clinicaltrials.gov);
5. Physiotherapy Evidence Database (PEDro), with no restriction regarding year;

All databases and trial registries will be searched from inception to date, and no restriction on language or type of publication will be applied. Grey literature will also be identified.

A search strategy with free and controlled terms about the lumbopelvic region will be established. The full search strategies can be found in S1 Appendix.

### Searching other resources

We will manually check the reference lists of all primary studies and review articles for additional references.

### Selection of studies

Two authors (SORL and C) will independently screen titles and abstracts of potential studies. Full text of potentially eligible studies will be retrieved and screened for inclusion. Reasons for exclusion of ineligible studies will also be recorded. Disagreements will be solved through discussion, or a third review author (ESRV) will be consulted if needed.

Duplicates will be identified and excluded, multiple reports of the same study will be grouped and the selection process will be detailed to complete the PRISMA flow diagram.

### Data extraction and management

Review authors will extract the following characteristics from included studies:

1. Methods: study design, total duration of intervention, details regarding any run-in period, number and location of study centers, study settings, withdrawals, and date of the study.
2. Participants: number of participants, mean age, age range, condition severity, diagnostic criteria, baseline lung function, smoking history, inclusion criteria, and exclusion criteria.
3. Interventions: intervention, comparison, concomitant medications, and excluded medications.
4. Outcomes: primary and secondary outcomes (specified and assessed) and time points reported.
5. Notes: funding of studies and notable conflicts of interest between authors.

Review author (SORL and VPSS) will independently extract outcome data from included studies. We will analyze if the “characteristics of included studies” table clearly reports outcome data. Disagreements will be solved by consensus or involving a third review author (ESRV). One review author (SORL) will transfer data to the Review Manager (RevMan 2014). Data in the systematic review will be double-checked with data from study reports. A second author (VPSS) will spot-check study characteristics for accuracy.

Authors of original studies will be contacted to provide further details if any information is unclear. Fluent individuals or Google Translate will translate studies published in other languages. Key results translated using Google Translate will be double-checked with our translators.

### Assessment of risk of bias in the included studies

Review authors (SORL and VPSS) will independently assess risk of bias in each study using the Cochrane Handbook for Systematic Reviews of Interventions [19]. Disagreements will be solved through consensus or involving a third review author (ESRV).

Risk of bias will be assessed according to the following domains:

Random sequence generation (selection bias): description of the method used to generate allocation sequence to verify whether it should produce comparable groups. Any important concerns about other possible sources of bias will be described and classified as low, high or unclear risk of bias.

Allocation concealment (selection bias): description of the method for allocation concealment before assignment, and assessment of whether allocation to intervention could have been foreseen before or during recruitment or changed after assignment.

Any important concerns about other possible sources of bias will be described and classified as low, high or unclear risk of bias.

Blinding of participants and personnel (performance bias): description of methods (if any) to blind study participants and personnel from knowing interventions received by participants. Low risk of bias will be considered if the study was blinded or if lack of blinding was judged unlikely to affect results. Blinding will be assessed separately for different outcomes or classes of outcomes, and methods will be determined as low, high or unclear risk of bias.

Blinding of outcome assessment (detection bias): description of methods (if any) to blind outcome assessors from knowing interventions received by participants. Blinding will be assessed separately for different outcomes or classes of outcomes. Methods to blind outcome assessment will be considered as low, high or unclear risk of bias attrition bias due to amount, nature, and handling of incomplete outcome data.

#### Selective reporting (reporting bias)

Description about how we will investigate possible selective outcome reporting biases. Will be described and classified as low, high or unclear risk of bias.

#### Other bias

Important concerns about other possible sources of bias will be described. Other biases will be evaluated as low, high, or unclear risk of bias.

### Assessment of quality of evidence

Quality of overall body of evidence for all primary outcomes will be assessed for all comparisons using the approach outlined in GRADE Handbook [20].

### Assessment of bias during the systematic review

The review will be conducted according to this protocol, and any adjustments will be justified in the “Differences between protocol and review” section of the systematic review.

### Measures of treatment effect

#### Dichotomous data

Results will be presented as summary risk ratios, with 95% confidence intervals for dichotomous data.

#### Continuous data

Mean differences of continuous data will be used if outcomes were measured using the same tools among studies. Standardized mean differences will be used for studies that measured the same outcome using different methods.

A consistent direction of the effect will be ensured if data from rating scales are combined in the meta-analysis (e.g., low scores always indicate improvement).

### Issues related to unit of analysis

We will consider participants rather than events as unit of analysis for dichotomous outcomes. Rate ratios reported in a study will also be analyzed using unit of analysis. The meta-analysis will be performed for cluster-RCT only if data was adjusted or could be adjusted.

### Dealing with missing data

Investigators or study sponsors will be contacted to verify key characteristics of the study and obtain missing numerical data when possible (e.g., when only the abstract is identified). Unsuccessful contacts and missing data introducing serious bias will also be considered for GRADE rating [20].

### Assessment of heterogeneity

The I^2^ statistic will measure heterogeneity among studies. If identified, substantial heterogeneity will be reported, and possible causes will be explored using a prespecified subgroup analysis.

### Assessment of reporting biases

Funnel plots will investigate reporting biases (e.g., publication bias) if ten or more studies are included in the meta-analysis. Funnel plot asymmetry will be visually evaluated, and exploratory analyses will be performed in the case of visual asymmetry.

### Data synthesis

A random-effects model and a sensitivity analysis with a fixed-effect model will be performed.

#### Subgroup analysis and investigation of heterogeneity

We plan to conduct the following subgroup analyses:

1. Gestational age;
2. Intervention duration.

The following outcomes will be used in subgroup analyses:

1. Pain intensity (pain level);
2. Low back or pelvic-related functional disability;
3. Functioning;
4. Quality of life.

Formal tests for subgroup interactions will be performed in the Review Manager (RevMan 2014).

### Sensitivity analysis

Sensitivity analyses will explore the influence of study quality on results. This will be assessed using allocation concealment or high attrition rates or both and excluding studies with high or unclear risk of bias from analyses to assess the difference in overall result.

## Discussion

As far as we explored, consensus or guidelines about the best modality or type, duration, and frequency of exercise for LPP during pregnancy are not yet available. Furthermore, previous reviews did not analyze the impact of these modalities considering functioning of pregnant women.

The publication of this protocol can aid other authors who are interested to review the impact of LPP on functionality, quality of life and aspects of exercise modalities protocols.

## Supporting information

S1 Checklist PRISMA-P (Preferred Reporting Items for Systematic review and Meta-Analysis Protocols) 2015 checklist: Recommended items to address in a systematic review protocol*. (DOC)

S1 Appendix (DOCX)
