# Supplementary material for: Therapeutic exercise versus other modalities for prevention and treatment of low back, pelvic girdle, and lumbopelvic pain during pregnancy: A review protocol
Source: PLoS One. 2022 Sep 22;17(9):e0274471. doi: 10.1371/journal.pone.0274471 (PMC9499307; doi:10.1371/journal.pone.0274471)
Supplement: S1 Appendix — (DOCX) [file pone.0274471.s002.docx]

**S1 Appendix**

Search strategy: Pubmed

(lumbopelvic pain) AND (pregnancy)

(lumbopelvic pain) AND (pregnant women)

((lumbopelvic pain) OR (low back pain)) AND (pregnancy)

(((lumbopelvic pain) OR (low back pain)) AND (pelvic girdle pain)) AND (pregnancy)

(low back pain) AND (pregnant women)

(pelvic girdle pain) AND (pregnant women)

(pelvic girdle pain) AND (pregnancy)

((lumbopelvic pain) OR (pelvic girdle pain)) AND (pregnancy)

((treatment) AND (lumbopelvic pain)) AND (pregnant women)

((treatment) AND (pelvic girdle pain)) AND (pregnancy)

((treatment) AND (pelvic girdle pain)) AND (pregnant women)

(((((treatment) ) AND (low back pain)) OR (pelvic girdle pain)) AND (pregnant)
